# Deciphering key genes in cardio-renal syndrome using network analysis

**DOI:** 10.6026/97320630017086

**Published:** 2021-01-31

**Authors:** Mohd Murshad Ahmed, Safia Tazyeen, Aftab Alam, Anam Farooqui, Rafat Ali, Nikhat Imam, Naaila Tamkeen, Shahnawaz Ali, Md Zubbair Malik, Romana Ishrat

**Affiliations:** 1 Centre for Interdisciplinary Research in Basic Sciences, Jamia Millia Islamia, New Delhi-110025, India; 2 School of Computational and Integrative Sciences, Jawaharlal Nehru University, New Delhi-1100067, India

**Keywords:** CRS, PPIN network, Module, Gene Ontology, Biomarkers, Pathways

## Abstract

Cardio-renal syndrome (CRS) is a rapidly recognized clinical entity which refers to the inextricably connection between heart and renal impairment, whereby abnormality to one organ directly promotes deterioration of the other one. Biological markers help to gain insight into the pathological processes for early diagnosis with higher accuracy of CRS using known clinical findings. Therefore, it is of interest to identify target genes in associated pathways implicated linked to CRS. Hence, 119 CRS genes were extracted from the literature to construct the PPIN network. We used the MCODE tool to generate modules from network so as to select the top 10 modules from 23 available modules. The modules were further analyzed to identify 12 essential genes in the network. These biomarkers are potential emerging tools for understanding the pathophysiologic mechanisms for the early diagnosis of CRS. Ontological analysis shows that they are rich in MF protease binding and endo-peptidase inhibitor activity. Thus, this data help increase our knowledge on CRS to improve clinical management of the disease.

## Background

The incidence of cardiac and chronic renal dysfunction gave a term Cardiorenal Syndrome (CRS), which has been widely used without well-known definition. It is classified within five subtypes and reflection of its pathophysiology includes time-frame, and combined cardiac-renal failure [1]. It can be commonly described as a pathophysiological disorder which shows strong connection in heart and kidneys whereby failure of one organ may induce acute or chronic dysfunction of another one [2]. Coexistence of heart-kidney dysfunction across multiple interfaces causes several complex disease conditions. Dysfunctional links of both organs makes it a more complex condition, so it needs a multidisciplinary health management system to optimize disease actual condition to diagnose, better treatment and to enhance patient outcomes as well [3]. Cardiac and renal both functions are essential for stable hemodynamic system, where neurohormonal mechanism plays important role in hemodynamic stability the mechanism involves autonomic nervous system, reninangiotensin- aldosterone system (RAAS), arginine vasopressin (AVP), and endothelin-1 (ET-1) [4]. Activation of Neurohormonal mechanism has important significance in the pathogenesis of CRS biomarkers that have clinical application. They had have potential of its diagnosis in evaluating HF with kidney malfunctioning and also provide prognostic value in CRS [5]. Biomarker areas are discussed encompassing both the heart and kidney. The pathophysiologic mechanism of heart and kidney dysfunction is yet to be completely defined. But it is well understood that a cardiac function decline causes a decrease in tissue perfusion and so it adversely affects renal perfusion, which leads to renal injury. Inter organ communication at molecular level induces vessel inflammation, cardiac fibrosis, atherosclerosis and hypertrophy which adversely affects cardiac and renal function [6]. Deep observation of associated CKD, CVD and its epidemiology, different etiologies of CRS make patient management a real challenge for physicians. Still there is no authentic therapeutic approach for CRS, due to the unique medical history of individual CRS patients, risk factors, response of specific treatment, and combined comorbidities [7]. The literature based data mining of key genes and their further gene ontology, pathways enrichment and complex protein-protein interaction analysis revealing these genes may be potent as biomarkers of CRS. They play a wide role in HF, unlike either acute or chronic renal failure. Biomarkers may have direct or indirect clinical relevance in order to diagnosis, optimization etc. Findings suggest that natriuretic peptides are the most well known biomarkers so far these are the base of both diagnosis and treatment [8]. Early stage diagnosis of renal dysfunction is difficult or almost impossible through the traditional markers, serum creatinine, though efforts to explore possible markers for initial detection of AKI are being made. It is possible that CRS associated biomarkers can become risk factors in an enhancement of clinical outcome of the disease. CVD has a higher rate of morbidity and mortality in patients with renal dysfunction, and its treatment can be modified according to cardiac biomarkers [9]. Moreover, natriuretic peptides that are developed as cardiac biomarkers, and many more novel biomarkers have been identified that are significant for CRS [10]. This study will summarize the litretreturs on newly developed biomarkers of renal and cardiac dysfunction and their vital roles, as well as effects of CRS. The aim of this study is to possibly compile the work published with the role of biomarkers in the pathophysiologic mechanism of CRS [11]. Through Protein-protein interaction network construction and their further analysis at different levels of the network using MCODE we found 12 promising genes. Gene ontology enrichment, transcription factor and pathway enrichment also searched for those genes to get deep insight. Protein interaction data reveals the functional interplay of currently reported new biomarkers relevant to renal and cardiovascular disease. The identified genes were found functionally involved in protease binding, regulation of blood vessel diameter, AGE-RAGE signaling pathway in diabetic complications, HIF-1 signaling pathway [12]. Novel biomarker candidates are currently reporting with astonishing speed, in particular facilitated by genomic and proteomic techniques. The biomarkers predictive in renal or heart disease also have therapeutic application for identifying heart dysfunction in renal diseases and renal injury in Heart disease. Thus, it may be employed in prognosis and guide therapy of CRS patients [13]. Therefore, it is of interest to identify target genes in associated pathways implicated linked to CRS. The results may provide information for further investigation of the mechanisms underlying CRS and for the development of potential treatment approaches.

## Methodology

### Literature search:

**Figure 1:**
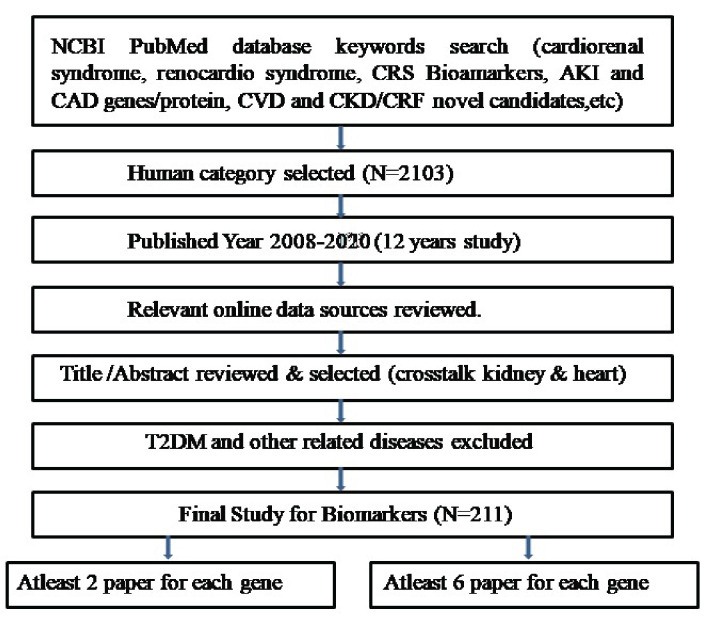
Flowchart of pubmed literature mining search strategy. A total of 104 studies were selected according to the inclusion criteria of this study.

The manual retrieval of key genes was done from the available resources like databases, literature relevant to CRS. Several important keywords was used throughout the search such as "cardiorenal syndrome", "renocardio syndrome", "Cardiovascular and chronic/acute kidney disease", "heart and kidney disease", "renal and myocardial/heart/congestive failure", "acute kidney injury and coronary artery disease", "CRS biomarkers", "CRS Type 1-5", "CKD and CVD", "AKI and MI", "CAD and CRF", "diagnosis" and "prognosis" in numerous databases and literature based search engine are PubMed database NCBI (National Center for Biotechnology Information https://www. ncbi.nlm.nih.gov/pubmed), Google Scholar and so on, and I came to find some potential studies published so far [14]. We customize the search options in order to filter articles only about humans, with specified duration of publication and in English language. Finally, 211 studies were evaluated through their titles and abstracts, and found irrelevant description, cross talk of other disease and exclusion of miRNA-mRNA drug response (according to my study). Resulted, 104 literatures were screened associated with Cardiorenal syndrome. Large number of articles revealed gene-disease association and further elucidation of biomarker genes may aid towards therapeutic options. Detailed description of literature mining is given in (Figure 1).

### Analysis and Establishment of Protein Protein Interaction Network (PPIN) construction:

The Protein-Protein Interaction Network (PPIN) was built by submitting the Cardiorenal syndrome associated genes in STRING version 9.1 databases (Search Tool for the Retrieval of Interacting Genes). It provides an integrative and critical assessment of PPI networks with the diversity of organisms. STRING is a biological database of known as well as predicted protein-protein interactions, that can offer deep insight of cellular processes [15]. The data integration weighted and confidence score of protein-protein interactions were calculated. Pairs of PPI nodes with Confidence score 0.4 was set as the threshold value. The PPI network constructed using STRING database further processed, visualized and analyzed.

### Module finding from MCODE:

For the cluster/module/community formation in the PPI network of genes, Cytoscape was used with plugin Molecular Complex Detection (MCODE), to identify highly interconnected regions in the network. The plugin provides tools for investigating and visualizing protein interactions on the detailed molecular level of modules and interaction sites. And default statistical parameters were used as "Degree cutoff = 2", "node score cutoff = 0.2", "k-core = 2 (default more than 1)" and "max. Depth = 100". The protein consisting modules involved cellular processes are functional while binding each other. Epochal modules can be identified as highly interconnected subgraphs and several computational approaches are now inevitable to extract from complex networks. Moreover, MCODE was employed to the highly dense modules at the level of network stability [16].

### Gene Ontology (GO) enrichment using Enrichr:

Gene ontology (GO terms) provides a dynamic, comprehensive, and standardized vocabulary that can be annotated in all eukaryotes, mostly including 3 independent categories, biological process (BP), molecular function (MF), and cellular components/localizations (CC). The Enrichr database (http://amp.pharm.mssm.edu/Enrichr/) was utilized to perform GO functional annotation P value < 0.05 was considered as statistically significant. Enrichment analysis is a popular method for analyzing gene sets generated by genome-wide experiments. Enrichr currently contains 332911 annotated gene sets from 164 gene set libraries. Enrichr, an integrative web-based and mobile software application that includes new gene-set libraries, an alternative approach to rank enriched terms, and various interactive visualization approaches to display enrichment results. The software can also be embedded into any tool that performs gene list analysis [17].

### Pathway enrichment analysis and transcription finding:

Pathway enrichment analysis (PEA) is a more potent approach for gene analysis in genomics, mostly applied to gene expression and support an interactive evaluation of the possible effects of variations on function, regulation or interaction of gene (mRNA) products. Genomic data are increasingly being used to identify biological pathways offers the potential of greater power for discovery with natural connections to biological mechanisms and networks underlying complex diseases [18]. Enrichr was continually enhanced with many new features, calculating the Fisher exact test by many folds so now the enrichment results are almost instant. We added a metadata term search function that allows users to fetch individual lists based on any search term that matches the gene set terms. The statistical significance of the enrichment was calculated using P-value < 0.01. Transcription factors (TFs) are proteins with DNA binding activity that is involved in the regulation of transcription. The ability to predict and identify TF binding sites throughout genomes is integral to understanding the details of gene regulation and for inferring regulatory networks [19].

## Results:

Peer-reviewed articles published in reputed journals were screened for key genes/proteins that are directly or indirectly associated with CRS. More than hundred (approx-104) publications published in between 2008 to 2020 covered a set of 119 non-redundant genes linked with CRS as described in Table 1 (see PDF) [20-117]. Out of them 54 CRS associated genes were reported in at least 5 articles. Biomarkers pertinent to the CVD and CKD interface are reported as cardiorenal/Reno cardiovascular biomarkers. Ahead, basic and clinical observations are claimed to elucidate the actual pathogenic role of increased NAGL and to validate the application of biomarkers for CRS. For a multi-biomarker approach a number of integrated assessments will be employed and shed light on the clinical management of CRS patients.

## Network Analysis and Module detection:

**Figure 2:**
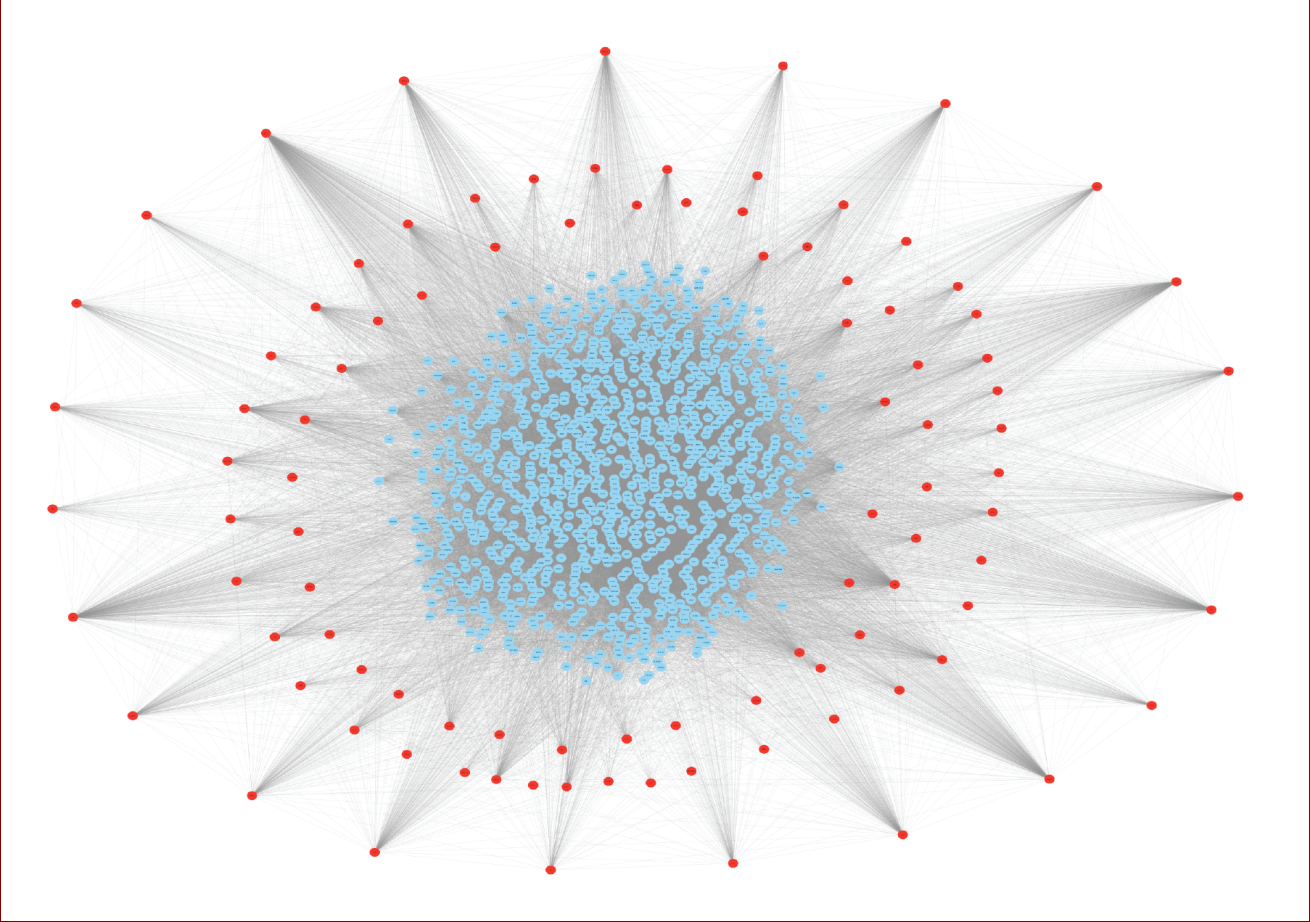
Protein - protein Interaction network containing 1149 nodes and 25452 edges. The red diamond indicates our seed genes and the blue circles are the interacting partners.

**Figure 3:**
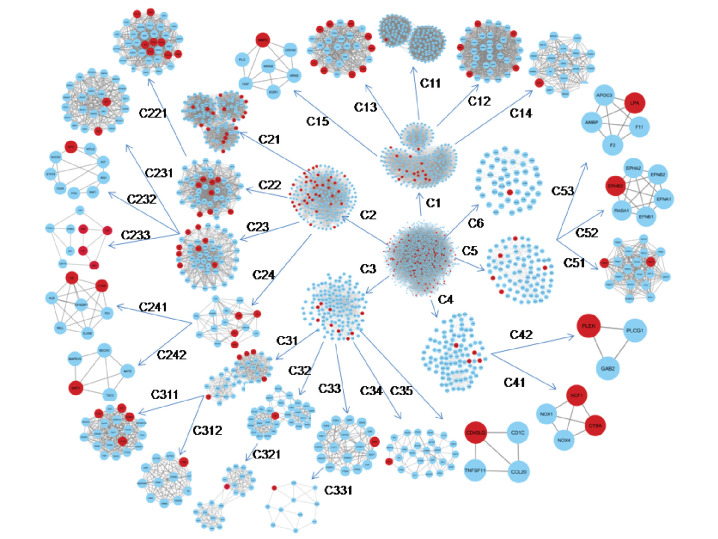
Module distribution at each level in which red color circles for our genes of interest.

Reported genes were used to make a PPI network, which was constructed using the STRING database, the network consisted of 1149 nodes and 25548 interaction pairs Figure 2. In that complex network top 10 scoring modules were identified via MCODE plug-in Cytoscape. Proteins in the network with similar functions tend to form clusters, and the function of a node is correlated with the distance between one node to another. Therefore, system level network analysis in current study allowed us to identify unknown functions of proteins. The highly interconnected regions in the PPI network were identified using MCODE plugin in Cytoscape, in the whole complex network 23 significant modules were formed and out of them we have chosen top 10 modules for further analysis. At the first level of network sub division six modules out of 10 having desired genes (seed genes) has been seen, only two submodules C2211 and C2331 at fourth level having 8 and 4 seed genes respectively as shown in Table 2. Interconnection among the modules represents the hierarchy nature of the network Figure 3andFigure 4. MCODE is a popular tool, it finds clusters within the network to do so it uses vertex weighting (a form of clustering coefficient) to form clusters from a vertex by iteratively adding neighboring vertices that have similar weight. Resulted, two clusters having 12 seed genes namely NOS3, EDN1, INS, TGFB1, EGFR, CCL2, SERPINE1, TIMP2, MPO, REN, CRP and ACE were traced at fourth level of network clustering.

## Gene Ontology (GO) functional Annotation, TFs and Pathway enrichment analysis:

The functions of clusters/subnetworks were evaluated by GO and pathway enrichment analyses (PEA). The results demonstrated that a total of 765 GO terms (MF and BP), 108 KEGG pathways and 114 TFs were enriched. Based on the most significant P values, the top fifteen GO terms, KEGG pathways and TFs were selected. For better understanding these 12 target genes, Enrichr database was utilized to perform GO function and KEGG pathway enrichment analysis. GO terms (MF and BP), KEGG pathways and TFs are described in Table 3 (see PDF), Table 4 (see PDF) and Table 5 (see PDF). Respectively. GO analysis indicated that the key genes were significantly enriched in protease binding, cytokine activity, mitogen-activated protein kinase kinase binding, hormone activity, transition metal ion binding, regulation of blood vessel diameter, regulation of cell migration, positive regulation of macromolecule biosynthetic process, negative regulation of blood vessel diameter, regulation of systemic arterial blood pressure by hormone, and Pathway information is inherently redundant, as genes often participate in multiple pathways, and databases may organize pathways hierarchically by including general and specific pathways with many shared genes, AGE-RAGE signaling pathway in diabetic complications, HIF-1 signaling pathway, Chagas disease (American trypanosomiasis), Relaxin signaling pathway, Renin secretion. Pathway enrichment analysis in to provide functional insight into the identified network marker. The interaction between transcription factor (TF) proteins and DNA is elementary to the regulation of transcription, a coordinated process that responds to environmental factors to achieve temporal and tissue specificity. Therefore, the ability to predict and identify TF binding sites throughout genomes is integral to understanding the details of gene regulation and for inferring regulatory networks. The NOS3 gene has many polymorphisms among them; the evidence showed that the coding region 4b/4a, the G894T, and the T786C variants is significantly implicated in CRS etiopathogenesis. Variations in the number of genes are the leading cause of increasing risk of the disease. A key gene plays a crucial role in the regulation of reduced blood vessel relaxation and NOS3; these are two main risk factors. REN genes found significantly involved in the progression of CV and disease independent of the classical renin-angiotensin-aldosterone-system. Downstream of ANG I and not targeting the activity of REN or their concentration, since the relation of heart failure and kidney problems with increased renin levels, mainly with the availability of ACEi or ARB. Loss of negative response of ANG II while renin release leads to growth of renin level secretion by ACEi and ARB. Analysis of multiple variations shows that greater levels of CRP significantly predict LV dysfunction and cardiac hypertrophy. Finally, increased levels of CRP in the patients having hemodialysis are linked with a higher risk of death that indicates some values towards prognosis for this inflammatory mediator. As a biological marker of systemic inflammation, CRP may play role in endothelial dysfunction by enhancing the expression level of endothelial cell adhesion molecules, lower level of nitric oxide and prostaglandin secretion from cells of endothelial tissues, augmenting low-density lipoprotein uptake by macrophages, and inducing complement-mediated inflammatory reaction. Chronically inhibiting the synthesis of NO may lead to upregulation of cardiac ACE and Ang II receptors, possibly mediating inflammatory changes. It has been previously reported that the stimulus for the sympathetic hyperactivity found in renal dysfunction generated from kidney failure and that growing sympathetic outflow in CRF possibly could be controlled with the inhibition of ACE. MPO plays a role as a master enzyme in the ROS generation through catalyzing the conversion of hydrogen peroxide. Increasing levels of MPO were able to observe higher risk of CAD development in normal candidates. MPO has been considered to be an important oxidative stress pathway in ESRD. Hypomethylation of EDN1 gene in collected duct cells of renal inner medullary that express high levels of ET-1. In contrast, fibroblasts have a hypermethylated edn1 gene and express only a lower amount of ET-1. In renal epithelial cells EDN1 gene has a major role for calcium, whereas Vezf1 has a special response of EDN1 gene activity in endothelial cells. Insulin resistance in endothelial cells promote the progression of prothrombotic factors level, proinflammatory markers, and ROS, that may cause to increase in the intracellular levels of adhesion molecule 1 (ICAM-1) and vascular cell adhesion molecule 1 (VCAM-1). In order to generate CVD two independent pathways play crucial roles through the contribution of Insulin resistance, (1) atheroma plaque formation and (2) ventricular hypertrophy and diastolic abnormality. Both pathways cause heart dysfunction. TGF-β1 found in both myocardial fibroblasts and cardiomyocytes. Higher expression of TGF-β1 is seen in heart during the process of cardiac development and pathology. Angiotensin II (Ang II) has potential for hypertrophic stimulus, responsible for progression of TGF-β1 gene expression. TGF-β1 is a principal mediator of the hypertrophic growth response of the heart to Ang II. Epidermal growth factor receptor (EGFR) (or ERBB1), a membrane tyrosine kinase receptor expressed in the kidney, showed the activity after renal failure, and many preclinical reports have shown it is a potent target in CKD therapy. Regulation of many cellular responses is associated with activity of EGFR signaling pathway, such as cell proliferation, inflammation, and regulation of extracellular matrix, all processes and mechanisms functionally involved in the onset and renal failure progression. CCL2 considerably reduces hypoxia-induced cell death in cultured cardiac myocytes. In all the modulating processes of chemokines, CCL2 and their receptor CCR2 found to be most important for the shift from physiological conditions to pathological conditions in both heart and vessels. CCL2 is chemotactically attractive for mononuclear cells that are a source of fibrogenic mediators like TGF-β and Fibroblast Growth Factor. Additionally, it causes synthesis of macrophages of both TGF-β1 and collagen. (SERPINE1), that has also been considered a mediator with potency of diabetic nephropathy and glomerulosclerosis. The study concluded that the cell cycle arrest biomarkers, urinary IGFBP-7, and TIMP-2, which may be responsive to take benefit towards clinical ways to predict the progression of type 1 CRS development after renal ischemia in patients having decompensated heart dysfunction. In this pilot study, ILGF-7 and TIMP-2 have been evaluated in the CRS development.

## Conclusion

We report 12 essential genes in the network through module analysis as potential emerging tools for understanding the pathophysiologic mechanisms for the early diagnosis of CRS. In this study we have established a complex potent CRS-related mRNA regulatory network, which gives a deep understanding of molecular mechanisms and it provides key points in seeking novel therapeutic options for CRS. Although the findings will be required to validate through experimentation. A computational system based approach provides a systematic framework to find a hope in terms of the connection of a single biomarker candidate towards driving functional dependencies between clinically interlinked diseases. With the gigantic amount of present data generated from heart and kidney clinical studies, a uniformed pipeline is required for data curation that will facilitate researchers in retrieval of potential biomarkers from existing literature. To deepen understanding of the genes mechanisms, functions and their involvement, Enrichr database search Gene Ontology and KEGG analysis was also performed. Our study shows a comprehensive picture of molecular features related to the functional interplay between renal and the cardiovascular system. In this study, a literature mining approach to curated genes reported in the context of the CRS, and analyzed their features on the level of protein interaction networks.

## Figures and Tables

**Figure 4:**
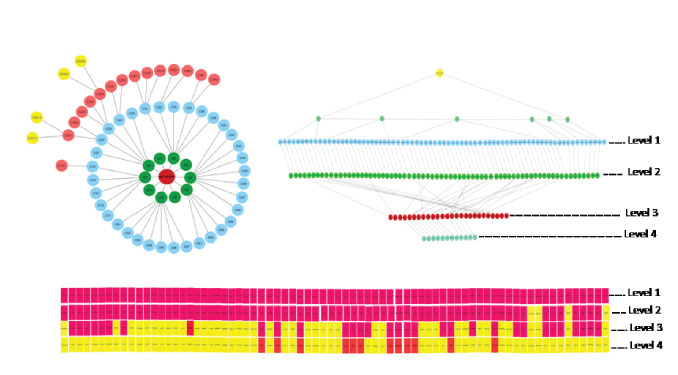
To illustrate community/module detection for gene tracing. (A) Top 10 module divided into sub network and further sub network break into sub network at the last level. (B) To shows how number of seed genes reduces from level 1 to level 4. (C) Heat map to shows the presence of our genes in each level whereas yellow color represents our genes absence.
